# Social prescribing for adults with chronic pain in the U.K.: a rapid review

**DOI:** 10.1177/20494637241312064

**Published:** 2025-01-21

**Authors:** Gerlinde Pilkington, Mark I. Johnson, Kate Thompson

**Affiliations:** Centre for Pain Research, School of Health, 4467 Leeds Beckett University, Leeds, UK

**Keywords:** Social prescribing, pain, voluntary, community and social enterprise sector, National Health Service, rapid review

## Abstract

**Introduction:**

Social prescribing links patients to community groups and services to meet health needs; however, it is uncertain what the benefits and impacts of social prescribing are for people with chronic pain. The National Institute for Health and Care Excellence (NICE) undertook a systematic review to investigate the clinical and cost effectiveness of social interventions aimed at improving the quality of life of people with chronic pain; no relevant clinical studies comparing social interventions with standard care for chronic pain were found, though the inclusion criteria for studies was narrow.

**Objectives:**

To undertake a rapid review of all types of research and policy on social prescribing for adults with chronic pain in the U.K. (i) to describe the characteristics of relevant research and (ii) to synthesise data on impact.

**Methods:**

A two-stage rapid review was planned. Stage (i) scoped and categorised knowledge from a comprehensive representation of the literature. In stage (ii), we undertook a descriptive synthesis of quantitative data along with a thematic analysis of qualitative data identified by stage (i).

**Results:**

Of 40 full-text records assessed for inclusion, three met the inclusion criteria from academic databases. An additional five records were found in grey literature. Six records reported quantitative findings suggesting that social prescribing reduced pain severity and discomfort, pain medication and clinical appointments; and improved quality of life and ability to manage health. Five records captured qualitative data from interviews, case studies and anecdotal quotes that suggested positive impact on health and wellbeing; and increased self-efficacy in social prescribers undertaking training on pain.

**Conclusions:**

There is tentative evidence that social prescribing improves health and wellbeing outcomes in adults with chronic pain and that there is a need to upskill social prescribers in contemporary pain science education. Research on the routes to referral, outcomes and impacts is needed.

**Perspective:**

Social prescribing is valued and may be of benefit for people with chronic pain. There is a need to further develop and evaluate social prescribing services for people with chronic pain to enhance holistic patient centered care.

## Introduction

Social prescribing is an approach that links people to a wide range of community groups and services to meet the practical, social and emotional needs that affect their health and wellbeing.^1^ There is no set definition of social prescribing; however, the Social Prescribing Network^2^ have formulated a good working definition:

‘A means of enabling GPs [General Practitioners] and other frontline healthcare professionals to refer patients to a link worker - to provide them with a face to face conversation during which they can learn about the possibilities and design their own personalised solutions, that is, “co-produce” their “social prescription” – so that people with social, emotional or practical needs are empowered to find solutions which will improve their health and wellbeing, often using services provided by the voluntary, community and social enterprise sector’ (p. 19).

In the U.K. National Health Service (NHS) context, people can be referred to non-clinical services through a link worker to engage in activities for social support and to improve wellbeing. Activities include yoga, pilates, art, dance, singing, knitting, cooking, sports, walking groups, and gardening clubs. Social prescribing may also link people to statutory services such as debt counselling, housing services and other agencies for practical and emotional support. Decisions on which group or service to access depend on the needs and desires of the individual.

Social prescribing has been shown to have an impact in several ways such as^3^: (p. 2)

• *Increased self-esteem and confidence, sense of control and empowerment*
• *Improved psychological or mental wellbeing*
• *Reduction in symptoms of anxiety and/or depression*
• *Improved physical health and a healthier lifestyle*
• *Increased sociability, communication skills and making social connections*
• *Reduction in social isolation and loneliness*
• *Improvements in motivation, meaning, hope and optimism*
• *Acquisition of learning, new skills and interests*

However, it is uncertain what the benefits and impacts of social prescribing are for people with chronic pain, which is defined as pain that persists or recurs for 3 months.^4^ The World Health Organisation (WHO) categorises chronic pain, into chronic primary pain (e.g. fibromyalgia, non-specific musculoskeletal pain, and irritable bowel syndrome) or chronic secondary pain (e.g. cancer-related pain, post-surgical or post-traumatic pain, secondary musculoskeletal pain, neuropathic pain, secondary headache, or orofacial pain).^5^

In 2021, the National Institute for Health and Care Excellence (NICE) commissioned an evidence review to evaluate social interventions for improving the quality of life of people with chronic pain. No randomised controlled trials (RCTs) or systematic reviews were eligible for inclusion resulting in an ‘empty review’.^6^ The research and policy landscape on social prescribing for chronic pain in the U.K. remains unknown, hindering the ability of policy-makers, clinicians, and patients to make informed decisions. We decided to conduct a rapid review; to map the knowledge base and identify gaps in the research literature, and to evaluate reported impact.^7–9^ The protocol is registered on PROSPERO (CRD42023442325).

## Methods

We planned to conduct a two-stage rapid review^7,8,10^ that involved (i) a mapping exercise to identify, categorise, and contextualise the knowledge base and (ii) a systematic review and meta-analysis of the impact of social prescribing, identified in stage (i). Unfortunately, the paucity of primary research precluded a meta-analysis being undertaken. Instead, we provide a descriptive synthesis of quantitative data and a thematic summary of qualitative data from records identified by the mapping exercise.

### Stage 1

We used standard methods for mapping to identify, categorise, and contextualise the knowledge base in five key steps: identifying the research question(s), identifying potentially relevant studies, selecting relevant studies, charting the data, and then collating, summarising, and reporting the data.^9,11^

#### Search sources

We conducted iterative searches to ensure a comprehensive representation of the literature up to 15^th^ July 2023.^11^ The following electronic databases were searched from 2013 to 15^th^ July 2023 using combinations of search terms identified in Supplemental File 1; MEDLINE, CINAHL, PsycINFO and Scopus. To identify relevant policy and guidance documents, we used keywords [‘social prescribing’ OR ‘social intervention’ OR ‘community intervention’] to search the websites of the Health and Care Professions Council (HCPC), British Pain Society, European Pain Federation (EFIC), International Association for the Study of Pain (IASP), Health Education England (HEE), Physiotherapy Pain Association (PPA), Royal Society for Public Health (RSPH), NHS England and the NICE (Supplemental file 1). We searched for grey literature using keyword searches in Google identified in Supplemental file 1.

#### Screening and selection

Titles and abstracts were downloaded to EndNote and deduplicated, then screened using Rayyan online software using an adapted PICO framework (Table 1). A random sample of 10% of all titles and abstracts were double-screened; there was at least 80% agreement between reviewers; therefore, the remaining titles and abstracts were single-screened. Full-text copies of potentially relevant records were retrieved and screened for inclusion using the population, concept, and context criteria outlined in Table 1. Any queries or disagreements were resolved by discussion, with a third reviewer being consulted where necessary.

**Table 1.** Eligibility criteria.

Population	Adults (aged ≥18 years) with chronic pain, defined as pain that persists or recurs for longer than 3 months
Concept	Studies of social prescribing interventions or community-based social intervention, either directly or via a link worker
	Qualitative and quantitative studies of any design which report any outcomes related to physical and mental health, for example
	• Health-related quality of life
	• Physical function
	• Psychosocial measures
	• Pain measures
	• Self-efficacy
	• Use of healthcare services
	Outcomes relating to the delivery of interventions such as attitudes, satisfaction, views, experience. Studies presenting sub-group findings for adults with chronic pain will be included. Editorial and opinion pieces will be excluded
Context	U.K.-based evidence

#### Mapping the data

Where reported, the following summary-level data were extracted into pre-designed and piloted forms: Author(s), Year of publication, Location, Aims/purpose, Study design, Population and sample size, Intervention description – method of referral, provider, activities, Outcome measures reported, Data relating to health inequities (e.g. ethnicity, gender, and socioeconomic status), and Summary of key findings. No formal assessment of the methodological quality of the included studies was conducted at this stage of the review.

### Stage 2

A descriptive synthesis of quantitative data, and thematic summary of qualitative data was undertaken by analysing and organising the findings of records identified by stage (i). After the quantitative data was extracted, it was organised according to outcome measure(s) used and impact on health and care outcomes. Qualitative data was inductively coded and analysed to construct summary themes.^12^

## Results

### Stage 1

#### Search results

**Figure 1.**
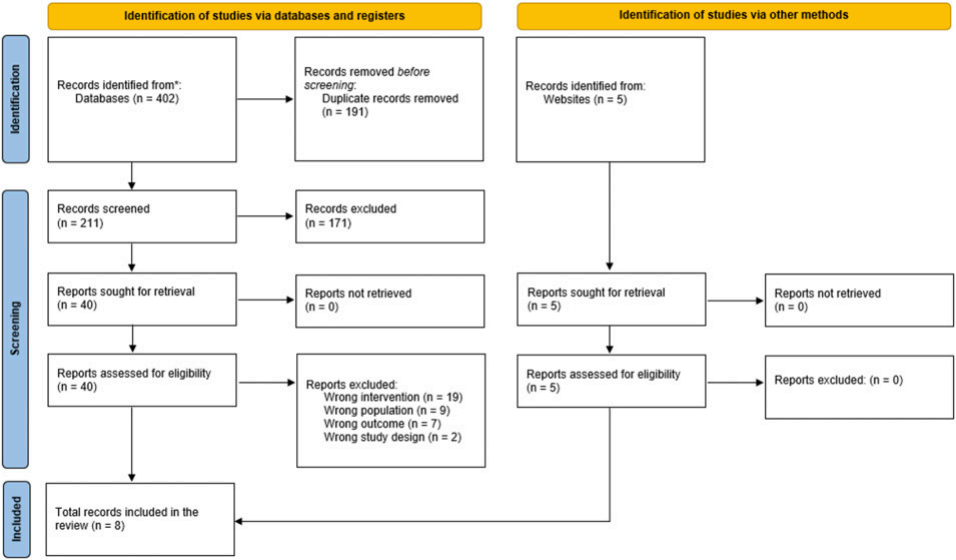
PRISMA flow diagram.

Searches of electronic databases yielded 402 records (Figure 1). After deduplication, 211 titles and abstracts were screened; of the 40 full-text records assessed for inclusion, three met the inclusion criteria.^13–15^ A further five records which met the inclusion criteria were found within the grey and unpublished literature.^16–20^ Hence, a total of eight records were included in the review (Table 2).

**Table 2.** The characteristics of included evidence.

Source of information	Record	Methods	Participants & Location	‘Interventions’	Outcomes
Academic database	Wright et al. (2017)	Mixed-methods evaluation	Adults with chronic pain	Kairos project – a charitable organisation delivering community-based interventions alongside an NHS pain clinic	Quality of life EQ5D questionnaire
			London, UK		Pain severity scores
					Number of outpatient appointments at NHS clinics (e.g. pain clinics, rheumatology, orthopaedics, and physiotherapy)
	Haake et al. (2022)	Survey	Parkrunners (varied health conditions)	Parkrun	Percentage of responders reporting improvements to managing their health condition
			UK		
	Corline et al. (2023)	Mixed-methods evaluation	Social prescribers	Pain education for social prescribers	Mean confidence scores
			Newcastle upon Tyne, UK		1-1 interviews
Grey literature	Dayson and Bashir (2014)	Mixed-methods evaluation	Service users (varied health conditions)	Social prescribing pilot in Rotherham	Hospital episode statistics – inpatient admissions, A&E attendances, outpatient appointments
			Rotherham, UK		Wellbeing measurement tool designed specifically for the programme, analysed against 8 outcome measures linked to wellbeing and functioning
					Interviews with patients and carers
					Case studies
					Economic and social impact – estimated NHS cost reductions
	Dayson (2018)	Mixed-methods evaluation	Service users (varied health conditions)	Community connectors social prescribing service	EQ5D questionnaire completed by service users with a variety of health conditions
			Bradford, UK		GP data
					Case studies
	Dayson and Leather (2020)	Mixed-methods evaluation	Service users (varied health conditions)	Community connectors social prescribing service	Community Connector monitoring data including
			Bradford, UK		- The Short Warwick-Edinburgh Wellbeing Scale (SWEMWBS)
					- EQ5D (health-related quality of life)
					Data from GP practice who made referrals
					Four service user case studies
	National Association of Primary Care (2017)	Mixed-methods	People who borrowed a ‘book on prescription’	Reading well books on prescription. Healthcare professionals, where appropriate, can prescribe self-help reading from approved lists of books available in public libraries	Survey of people using ‘books on prescription’
			Library staff		Survey of participating library authorities
			UK		Survey of ‘prescribing partners’, that is, health practitioners
			Healthcare partners		Qualitative interviews with library staff and healthcare practitioners
	Sheffield Clinical Commissioning Group (2019)	Unclear	Service users (varied health conditions)	‘People Keeping well in their community’ – social prescribing service	Anecdotal quotes from people who accessed the service
			Sheffield, UK		

All evidence provided by included records is U.K.-based. Five records reported data from the north of England, including Newcastle upon Tyne,^15^ Rotherham,^17^ Bradford,^16,18^ and Sheffield,.^20^ One record reported data from London^13^ and two were U.K-wide.^19,21^ Table 2 summarises the characteristics of the evidence. A short description is provided here.

Three records were located in peer review journals^13–15^ Wright et al. (2017)^13^ undertook a mixed-methods evaluation of a community-based rehabilitation and social intervention programme for patients with chronic pain with associated multi-morbidity and found improvements in chronic pain related disability and reduced healthcare use. Haake et al. (2022)^14^ undertook a secondary analysis of an online survey of parkrunners in the U.K.; a total of 66.8% of respondents reported improvements to ‘your ability to manage your health condition, disability, or illness’. Respondents had a range of health conditions including arthritis, chronic pain and fibromyalgia. Corline et al. (2023)^15^ investigated the impact of education for social prescribers, reporting that training led to improvements in self-reported confidence of social prescribers in supporting self-management with people experiencing chronic pain.

Five records were located in the grey literature.^16–20^ Dayson and Bashir (2014)^17^ undertook a mixed-methods evaluation of a social prescribing pilot in Rotherham, U.K. reporting some improvement in ‘managing symptoms’ (including pain and discomfort) and health and wellbeing. Dayson (2018)^16^ and Dayson and Leather (2020)^18^ undertook a mixed-methods evaluation of a social prescribing service in Bradford, U.K. There are two records by Dayson and colleagues including a mid^16^ and end of year evaluation of the service.^18^ The record relating to the mid evaluation reports improvement in pain and discomfort as part of a health-related quality of life measure.^16^ The end of evaluation report reiterates the findings of the mid evaluation report along with evidence from case studies demonstrating improvements in pain, discomfort, health and wellbeing outcomes.^18^ The National Association of Primary Care (2017)^19^ published a descriptive report with some evaluation findings of the ‘Reading Well Books on Prescription Scheme’. The evaluation findings include verbatim quotes from a pain nurse who describes positive outcome in health and wellbeing from a client with chronic pain. Sheffield Clinical Commissioning Group (2019)^20^ produced a briefing note for GPs about a local social prescribing initiative called ‘People Keeping Well in their Community’. Anecdotal quotes from patients who participated in a Chronic Pain Group, as a part of the social prescribing initiative, suggest improvements in pain and self-confidence.

### Stage 2

#### Outcomes and impacts

A descriptive synthesis of quantitative findings and a thematic analysis of qualitative findings of records located by the mapping exercise is presented here. The outcomes and impacts reported in each record is summarised in Supplemental File 2.

#### Quantitative findings

The quantitative measures reported within the records located by this rapid review were heterogenous and there was insufficient data to undertake a meta-analysis. Quantitative measures used to evaluate service user or patient outcomes included: EQ5D quality of life,^13,16,18^ pain severity scores,^13^ number of healthcare appointments,^13,16–18^ self-reported improvements in managing own health condition,^14^ and wellbeing measurement tools/scales.^17,18^ One record reported the mean confidence of social prescribers in supporting people with chronic pain after a chronic pain training programme.^15^

Wright et al. (2017)^13^ reported various beneficial outcomes associated with community-based interventions alongside an NHS pain clinic including; improvement in median EQ5D (quality of life) scores from 0.23 to 0.329, a reduction in median pain severity scores and repeat prescribing for pain, a 51% decrease in specialist outpatient appointments (including rheumatology, orthopaedics, neurology and neurosurgery) and an 86% decrease in the total number of appointments attended for psychology, physiotherapy, podiatry, acupuncture and cognitive behavioural therapy.^13^ Haake et al. (2022)^14^ did not specifically report outcomes related to pain because survey respondents had a variety of health conditions, although they found that 66.8% of respondents reported improvements to ‘your ability to manage your health condition, disability, or illness’.^14^ Dayson and Bashir (2014)^17^ reported that 21% of participants made progress in managing symptoms, of which pain is included but not itemised, at 4 months follow-up. Of the patients with a low baseline score, 57% made progress.^17^ In the mid evaluation report, Dayson^16^ states that the overall improvement in ‘pain and discomfort’ was 27% with the largest reduction being in the number of service users with severe or extreme pain and discomfort (11% improvement). In the end of evaluation report, Dayson and Leather^18^ state that the overall improvement in pain and discomfort was 14%, with a 2% improvement in pain and discomfort for those reporting severe or extreme problems.

#### Qualitative findings

Qualitative outcomes were predominantly captured from interviews,^15,17^ case studies^17,18^ and anecdotal quotes.^19,20^ Qualitative data mostly reported the impact of social prescribing on various health and wellbeing outcomes for people experiencing chronic pain.^17–20^ One record^15^ reported qualitative data in relation to social prescribers experience of chronic pain training. Following inductive coding, three themes were constructed to summarise the findings relative to three topics^12^ :

##### Impact on quality of life

This theme includes qualitative data that relates to the impact of social prescribing on physical and/or social and/or mental health for people living with chronic pain. There is an emphasis on considering factors beyond the biomedical model and the perceived value of a holistic approach to pain management. The quotes from social prescribers suggest that social prescribing has the potential to improve quality of life, with multiple aspects of health and wellbeing addressed.

Dayson and Bashir (2014) “*She has regained some independence, and feels better physically and emotionally because she has something to look forward to. Without the Social Prescribing service, she would withdraw within herself and become isolated again.”*^17^(p. 50)

Dayson and Leather (2020) “*She said she thought it helped because she did not feel alone anymore. She made new friends and looked forward to going and socialising as well as participating in the support group. Nazmeen reported that the support given by her HALE [Healthy Action Local Engagement] Community Connector excellent and really appreciated the help to move forward*s.”^18^(p. 16)

NAPC (2017) “*One patient who accessed Overcoming Chronic Pain said she found it helpful and commented that she is now aware that her pain ‘may well get worse but won’t kill’ her .. She understands that exercise is safe and requested details about a ‘sensible exercise’ programme*.”^19^(p. 20)

##### Alleviation of pain and discomfort

This theme includes qualitative data that reported the impact of social prescribing on physical symptoms of pain and/or discomfort for people living with chronic pain. The data tentatively suggests that social interventions may alleviate pain and discomfort for some individuals, from the perspective of a social prescriber and patient.

Dayson and Bashir (2014) “ … *She went to an exercise class and Mrs D feels she benefited from it immensely, for example, her shoulder pain has gone and she has started walking with ease, noting ‘a big difference’*.^17^(p. 50)

Sheffield Clinical Commissioning Group (2019) *“My sciatica has been eradicated; I couldn’t stand for even 5 minutes but now can walk for 1.5 hours a week*.”^20^(p. 5)

##### Impact on confidence/self-efficacy

There are two sub-themes within this theme. Theme 3[a] relates to the impact of social interventions on the confidence and self-efficacy of people living with chronic pain. Theme 3[b] relates to the impact of pain training on the confidence of social prescribers in supporting people with chronic pain.

##### Impact of social prescribing on confidence/self-efficacy of people with chronic pain

Data within this theme highlights the perceived change in confidence or self-efficacy from the perspective of social prescribers as a result of finding support, understanding and company through social interventions.

Dayson and Leather (2020) “*Nazmeen is now supporting a new fibromyalgia group set up by HALE [Healthy Action Local Engagement] and Champions Show the Way as a volunteer. Nazmeen feels like she is giving back to the community after all the support she received and says that volunteering has improved her self-confidence, self-esteem and life satisfaction.”*^18^(p. 16)

NAPC (2017) “*Before being prescribed the book, this lady had a very medical focus; she wanted surgery. She now believes that she can develop self-management skills without having to rely on the NHS*.”^19^(p. 20)

##### Impact^20^ of pain education on confidence/self-efficacy of social prescribers to support patients

Of note, Corline et al. (2023)^15^ highlight the lack of support and training that social prescribers and link workers receive to support people living with chronic pain, and how training in the Ten Footsteps to pain self-management programme (https://livewellwithpain.co.uk/ten-footsteps-programme/) has increased social prescriber and link worker confidence. There was an element of ‘surprise’ in the voice of social prescribers at the value that they experience in the training and how they could apply it to their practice.

*“I can’t understand why everybody isn’t given this training.”*^15^(p. 286)

*“This is the first time I’ve received any resources [about self-management]. No, I’ve never had anything like this before.”*^15^(p. 286)

*“[I would] explore how different factors can affect somebody’s pain and how looking at the “medical model” solely isn’t going to make a difference if somebody’s got persistent pain (…) people have, I supposed listened to it more knowing that they’ve (…) got medical support alongside [self-management*].*”*^15^(p. 287)

Health care staff reported that the Reading Well Books on Prescription scheme had positive impact on their confidence/self-efficacy to support people living with chronic pain:^19^

NAPC (2017) *“I am thrilled we tried it. Reading Well Books on prescription is currently used by the two pain nurses and our database shows that over 6 months we have issued more than 40 prescriptions*.”^19^(p. 20)

#### Current policy and guidance recommendations

For the most part, policy and guidance documents relating to social prescribing located by our search, focused on workforce development of social prescribers and link workers in the NHS, or social prescribing in general, rather than for chronic pain. For example, The NHS Long Term Plan^22^ sets out social prescribing within a desire to deliver more person-centred care, but makes no reference of how this specifically relates to chronic pain:

NHS (2019) *“…the range of support available to people will widen, diversify and become accessible across the country. Link workers within primary care networks will work with people to develop tailored plans and connect them to local groups and support services. Over 1,000 trained social prescribing link workers will be in place by the end of 2020/21 rising further by 2023/24, with the aim that over 900,000 people are able to be referred to social prescribing schemes by then.*”^22^(p. 25)

The NHS Digital Data for Primary Care Network Workforce report for England demonstrates that the number of Social Prescribing Link Workers is rising, with 2791 full time equivalent in employment and 1011 Health and Wellbeing Coaches as of September 2023.^23^

In terms of management of chronic pain, NICE guideline NG193/7^24^ was unable to recommend social interventions for the management of chronic pain due to a lack of evidence. However, the committee noted that:

The NICE (2021) *“…provision of social prescribing link workers is part of the NHS long term plan, and so there is already a move towards social interventions within the NHS. The committee were aware of evidence for social interventions in conditions other than chronic pain, but they agreed that this evidence could not be extrapolated as the issues faced by people with chronic pain are likely to be different from those populations. They could not make a recommendation for chronic pain without evidence on clinical and cost effectiveness. The committee decided to make a research recommendation to gather high-quality evidence on social interventions in the NHS, specifically for adults with chronic pain. This will hopefully inform future guidance.”*^6^(p. 38)

Guidance for Allied Health Professionals (AHPs) is set out in ‘Driving forward social prescribing: A framework for Allied Health Professionals’.^25^ The framework is not specific to chronic pain, however there are ideas for AHPs to implement social interventions, along with case examples of signposting by physiotherapists, osteopaths, occupational therapists, and radiographers, for patients with pain or chronic pain.

Although many people with chronic pain may be encouraged to participate and benefit from social prescribing, there is a lack of guidance for health care professionals, social prescribers, and link workers which specifically relates to social prescribing for people with chronic pain.

### Summary of evidence

The extent and nature of the knowledge base for social prescribing for adults with chronic pain in the U.K. appears sparse. In the records located by this rapid review, data was predominantly collected by mixed-methods, including case studies, interviews, surveys, and quality of life measures focussing on health and wellbeing outcomes for people living with chronic pain. One record reported the impact of pain training on improving confidence of social prescribers supporting people with chronic pain. Findings offer no more than initial insight to the possibility of social prescribing providing benefits for the health and wellbeing of people living with chronic pain. We did not find any policy documents that directly inform social prescribing for people with chronic pain. Recommendations for social prescribing more generally, may be transferable to chronic pain.

## Discussion

This rapid review found that the extent and nature of the knowledge base for social prescribing for adults with chronic pain in the U.K. was sparse, and that current policy and guidance recommendations for the provision of social prescribing for adults is general rather than being tailored to the specific needs of people with chronic pain. We found a paucity of research evidence to judge the impact of social prescribing for people living with chronic pain, with only three small studies located in academic databases, two service evaluations (of which one produced two reports) and two clinical reports/briefing notes. There was insufficient high-quality evidence to judge the benefits or harms of social prescribing for chronic pain, a finding consistent with the NICE evaluation published in 2021.^24^ Evidence presented in our review is at best ‘tentative’, suggesting that social prescribing may help people with chronic pain by reducing pain severity and discomfort,^13,17,18,20^ improving health and wellbeing,^17–19^ improving health-related quality of life,^18^ improving health self-efficacy or confidence,^18–20^ reducing prescription of pain and other medications,^13^ reducing clinical appointments,^13^ and improving the ability to manage one’s own health.^14^

When searching for information, we found that most social prescribing research had focussed on mental health, loneliness, and isolation. The paucity of research on the impact of social prescribing on health and wellbeing outcomes for people living with chronic pain may be because such people are not always seen as a distinct group of patients, but rather chronic pain is a symptom of other health conditions (e.g. diabetes and arthritis); thus, pain may not be ‘logged’ as the principal diagnosis when accessing social prescribing. However, this may change as chronic primary pain is now categorised in the International Classification of Disease (ICD-11).^5^ Unlike secondary pain, which can be attributed to a specific injury, disease, or condition, chronic primary pain is considered a primary health condition in its own right.^5^ It is likely that social prescribing and/or social interventions are being used for people with chronic pain in the U.K., but these were not found by our search strategy that focussed on identifying research and policy literature. Thus, a map of national social prescribing activity for chronic pain is needed.

We advocate for increased research activity to explore the impact of social prescribing and/or social interventions on an individual and population level, using mixed methodologies. We need research that evaluates benefits for conventional outcomes for biopsychosocial indices of health and wellbeing including pain, function, biomedical variables, and quality of life, as well as harms. In addition, we advocate research that investigates the living experience of receiving social prescribing through a cultural, socioeconomic, or environmental lens, considering how social prescribing sits within ‘personalised care’.

The lack of policy to directly inform social prescribing pathways for people with chronic pain may be a factor in why this area of practice and research is poorly represented in the literature. For instance, when reviewing social prescribing research, policy, and grey literature, it is apparent that social prescribing is implemented differently across different geographical areas of the U.K. There has been increased funding for personalised care since the passage of the Health and Care Act (2022),^26^ with increased social prescribing embedded in primary care. However, despite Clinical Commissioning Groups (CCGs) and NHS Trusts widely advertising social prescribing for people with chronic pain, there is a lack of guidance and/or training for social prescribers specifically about chronic pain. We argue that social prescribers have a critical role in helping to address health inequalities and support people experiencing chronic pain, particularly because the prevalence and impact of chronic pain is known to be unequal between different socioeconomic areas,^27^ age, gender, and ethnic groups.^28^ This as a critical area in which to conduct further research because, as social prescribing expands through NHS services, a need will arise to upskill social prescribers with knowledge and understanding of contemporary pain science through education. Misunderstanding the nature of chronic pain is common, and especially chronic *primary* pain where the underlying pathology of pain is unclear and/or its impact is out of proportion to any observable injury or disease. This may result in social prescribers inappropriately referring people with chronic primary pain into biomedical pathways of care. Corline et al. (2023),^15^ included in our rapid review, found that social prescribers participating in pain science education reported that their confidence to support people self-manage pain improved, and that pain science education aligns with the social prescriber role. Longer-term research will be important to determine how social prescribers are able to integrate these skills within their roles for the benefit of people with chronic pain.

### Recommendations for researchers, clinicians, and policy makers

Our rapid review supports a need for research, using mixed-method approaches, to understand not only the effectiveness of social prescribing but also the impact of social prescribing on an individual’s living experience. In this regard, we advocate that clinicians negotiate goals that are meaningful in the context of the person living with chronic pain when evaluating the impact of social prescribing because standardised health and wellbeing measures may not be as meaningful or important to the individual in some circumstances. Two studies included in our rapid review^13,18^ used the EQ5D to measure quality of life,^29^ and this may be an appropriate tool for measuring service impact and commissioning. Likewise, we advocate for policy to guide social prescribing pathways for people with chronic pain, considering holistic person-centred approaches that include clinical pathways connecting individuals into community and support networks that are culturally relevant and appropriate.

### Strengths and limitations

This rapid review mapped the nature and extent of knowledge to reveal a paucity of research evidence in a timely manner. We acknowledge shortcomings common to rapid reviews may introduce limitations and bias associated with shortcuts and omissions in the review process that undermines the certainty and confidence of the interpretation of the findings. The search used a limited number of databases and was not as comprehensive as a full scoping review, although we are confident that the search strategy and screening process was sufficiently robust to capture the majority of available evidence. We utilised three reviewers, although a single reviewer conducted some steps, and this has potential to introduce errors and/or bias in the selection process. The paucity of records included in the review means that the impact of this on our interpretation of the findings is likely to be negligible.

## Conclusion

In conclusion, this rapid review has shown that there is a paucity of evidence on which to judge the benefits, harms or impact of social prescribing specifically for people with chronic pain, including routes to referral and outcomes and experiences of participating in social prescribing schemes. Many of the records assessed for inclusion were excluded because the participants were inadequately described in terms of reasons for referral. In many cases where participants were described and people with chronic pain were included, outcomes and impacts were not discussed or reported by reason for referral. Future research should be more explicit about who participates and why, and to present findings accordingly.

## Supplemental Material

Supplemental Material - Social prescribing for adults with chronic pain in the U.K.: a rapid review Supplemental Material for Social prescribing for adults with chronic pain in the U.K.: a rapid review by Gerlinde Pilkington, Mark I. Johnson and Kate Thompson in British Journal of Pain
