# Supplementary material for: Social prescribing for adults with chronic pain in the U.K.: a rapid review
Source: Br J Pain. 2025 Jan 21;19(3):176–86. doi: 10.1177/20494637241312064 (PMC11752153; doi:10.1177/20494637241312064)
Supplement: Supplemental Material - Social prescribing for adults with chronic pain in the U.K.: a rapid review [file sj-pdf-1-bjp-10.1177_20494637241312064.pdf]

Supplemental file 1. Search strategy

ORIGINAL SEARCH STRATEGY/RESULTS

“social intervention” OR “community intervention” OR “wellbeing programme*” OR “well-being programme*” OR “well-being refer*” OR “wellbeing refer*” OR “referral scheme*” OR “link work*” OR “community refer*” OR “social refer*” OR “social prescri*” OR “community prescri*”

AND

Pain

CINAHL	28
MEDLINE	32
PSYCINFO	38
SCOPUS	106
**TOTAL**	**204**
**After deduplication**	**165**

SECONDARY SEARCHES

"social prescribing" OR "social intervention" OR "community assets" OR "arts on prescription" OR "education on prescription" OR "books on prescription" OR "exercise on prescription" OR "museums on prescription" OR "Information on prescription" OR "supported referral" OR "green gym"

AND

arthritis OR diabetes OR fibromyalgia OR "back pain" OR "irritable bowel" OR "chronic pain" OR "persistent pain" OR pain

CINAHL	44
MEDLINE	41
PSYCINFO	20
SCOPUS	93

**TOTAL**	**198**
**After deduplication**	**91**

POLICY

Keyword searches “social prescribing” OR “social intervention” OR “community intervention” OR “link worker”

Health and Care Professions Council (HCPC)	0	
British Pain Society	0	
European Pain Federation (EFIC)	0	
International Association Study of Pain (IASP)	1	• Research paper already identified by database search (Corline et al)
Health Education England	1	• Social prescribing at a glance
Physiotherapy Pain Association (PPA)	1	• Social Prescribing: A journey in South Tyneside
Royal Society for Public Health (RSPH)	5	• Project report for: Driving forward social prescribing A framework for Allied Health Professionals<br>**FRAMEWORK PDFs:**<br>• Active signposting<br>• Refer to a link worker<br>• AHP as social prescriber<br>• AHPs promoting, growing and developing social prescribing
NHS England	2	• NHS Long Term Plan<br>• Workforce development framework: social prescribing link workers
NICE	2	• Chronic pain (primary and secondary) in over 16s: assessment of all chronic pain and management of chronic

		primary pain<br>• Visual summary

GREY LITERATURE

Google:

(“social prescribing” | "social intervention" | "community assets" | "arts on prescription" | "education on prescription" | "books on prescription" | "exercise on prescription" | "museums on prescription" | "Information on prescription" | "supported referral" | "green gym") (“chronic pain” | “persistent pain” | pain)

https://www.google.co.uk/search?as_q=Google%3A+%28%E2%80%9Csocial+prescribing%E2%80%9D+%7C+%22social+intervention%22+%7C+%22community+assets%22+%7C+%22arts+on+prescription%22+%7C+%22education+on+prescription%22+%7C+%22books+on+prescription%22+%7C+%22exercise+on+prescription%22+%7C+%22museums+on+prescription%22+%7C+%22Information+on+prescription%22+%7C+%22supported+referral%22+%7C+%22green+gym%22%29+%28%E2%80%9Cchronic+pain%E2%80%9D+%7C+%E2%80%9Cpersistent+pain%E2%80%9D+%7C+pain%29&as_epq=&as_oq=&as_eq=&as_nlo=&as_nhi=&lr=lang_en&cr=countryGB&as_qdr=all&as_sitesearch=&as_occt=any&as_filetype=&tbs=

Supplemental file 2: Outcomes and Impacts of Social Prescribing and Chronic Pain

Reference	Quantitative	Qualitative
Wright et al., (2017) [1]	Before starting the Kairos Project (KP) the median EQ-5D index value of health status was 0.23 (IQR 0.294), compared with 0.329 (IQR 0.483) by the end of the project.<br><br>There was a reduction in participants’ median pain severity scores for least, average and worst pain, and in pain interference in most domains (activity, walking ability, work, relationships, sleep and life), after attending the KP.<br><br>There was reduction in the number of patients reporting	Not reported

	impairment across the EQ5D life quality domains after participation in the Kairos Project. Attending the project reduced the number of people reporting severe impairment in the Usual Activity, Pain and Anxiety/Depression domains.<br><br>Data on medication prescribed for pain before and after participation was available in 23 patients. There was a marked reduction in repeat prescribing for both number of items of medication and cost between initial referral to the KP and post discharge from the KP (average of 19 months). This was prescribed analgesic and psychotropic medication and did not include over the counter medication taken by patients.<br><br>The number of specialist out-patient appointments attended in secondary care pain clinics or in specialities related to pain management (rheumatology, orthopaedics, neurology, neurosurgery) fell from 68 in the 12 months preceding participation in the Kairos Project to 33 in the 12 months prior to July 2015 which was on average 19 months after the last contact with the KP in the 23 patient sample. This represents a 51% drop. There was an 86% drop in the total number of appointments attended for psychology, physiotherapy, podiatry, acupuncture and cognitive behavioural therapy (CBT) from 208 to 30.	
Haake et al., (2022) [2]	There were a paucity of outcomes specific to chronic pain<br><br>A total of 66.8% of all responders reported improvements to 'your ability to manage your health condition, disability, or illness' in response to an online survey question	Not reported
Corline et al., (2023) [3]<br><br>'Power to the People, to the people': Training for social prescribers improves support of	A total of 53 participants answered the pre-trial questionnaire and 25 answered the three-month follow-up. Respondents were 31 social prescribers, 17 wellbeing coaches, 3 general practitioners, one paramedic and one pharmacist.	'I can't understand why everybody isn't given this training' [Participant 2, Social Prescribing Coordinator, new to working with persistent pain]<br><br>'This is the first time I've received any resources [about self-management]. No, I've never had anything like this before' [Participant 5 Health Connector, experienced]

persistent pain	Mean confidence scores of the 10 self-management domains (Footsteps) were significantly different between baseline (M = 3.02, SD = 0.79) and 3-month follow-up (M = 4.06, SD = 0.56), t(20) = 5.012, p < .001. Cohen's effect size value (d = 0.951), 95% CI [0.6, 1.47] was large.	'[I would] explore how different factors can affect somebody's pain and how looking at the "medical model" solely isn't going to make a difference if somebody's got persistent pain (…) people have, I supposed listened to it more knowing that they've (…) got medical support alongside [self-management]'. [Participant 7, Social Prescriber, new to working with persistent pain]<br><br>'I think they definitely see pain as a medical issue. So they know that their lack of exercise or activity is increasing their pain, but I don't think they would look at diet, sleep, you know? They would see those as separate. I think it all stems from that'. [Participant 6, Social Prescriber, new to working with persistent pain]
Dayson and Bashir (2014) [4]<br><br>The social and economic impact of the Rother Social Prescribing Pilot	Baseline data was collected from 819 patients, 280 patients were followed up after 3-4 months through a well-being measurement tool developed specifically for the service. The quantitative data analysis is based on the analysis of the 280 patients with follow up data.<br><br>In regards to managing symptoms (of which pain is included but not itemised), after 4 months follow up, 21% made progress.Of the patients with a low baseline score, 57% made progress.	Mrs A is 56 years old and lives at home with her daughter, daughter-in-law and two grandchildren. She is able enough to take care of herself, but has increasingly experienced joint pain from arthritis over the last two-three years.<br><br>Mrs A feels very down at night when the arthritic pain is at its worstMrs A reported that previous to assistance from Tassibee '*I was completely stuck*, *I didn't know who to go to and what to do'*. Mrs A reports that she feels a little better within herself after assistance from Tassibee.<br><br>Mrs D is 72 years old and lives with her son and family. Mrs D has arthritis, a visual impairment and various other health problems. Prior to Tassibee assistance, Mrs D reported that she felt locked in her home. Since Tassibee assistance she reported that<br><br>*'Getting out has made me feel very good'*. She went to an exercise class and feels she benefited from it immensely, for example, her shoulder pain has gone and she has started walking with ease, noting '*a big difference'*. She has regained some independence, and feels better physically and emotionally.
Dayson (2018) [5]	12% of service users referred to the service with a long term health condition experienced chronic pain.<br><br>Service users' average score (overall health) on the EQ-5D improved from 37-47 (0-100mm VAS scale) following	

	referral to a community connector.<br><br>In terms of specific elements of health related quality of life (HRQL), improvements were most pronounced for anxiety and depression (36 per cent of service users improved), followed by usual activities (29 per cent improved) and pain and discomfort (27 per cent improved).<br><br>For those service users who reported severe or extreme problems for each measure: the largest reduction was in the number of service users with severe or extreme pain and discomfort (11 per cent) followed by anxiety and depression (10 per cent) and usual activities (8 per cent).	
Dayson and Leather (2020) [6]	\Health related quality of life was measured using the EQ-5D measure<br><br>Improvements were most pronounced for anxiety and depression (39 per cent of service users improved), followed by usual activities (20 per cent improved) and pain and discomfort (14 per cent improved).<br><br>For those service users who reported severe or extreme problems for each measure (figure 9): the largest reduction was in the number of service users with a severe or extreme score anxiety and depression (14 per cent) followed by usual activities and self-care (both 3 per cent) and severe or extreme pain and discomfort and mobility (both 2 per cent).	Case study E: Nazmeen is a 56 year-old British Pakistani with fibromyalgia who was referred to Community Connectors by her GP. Nazmeen's condition meant that she was in a lot of pain for a lot of the time and suffered from severe headaches.<br><br>Immediate impact: Nazmeen began attending the Support Group regularly and felt that they understood what she was going though. Nazmeen said she thought it helped because she did not feel alone anymore. Nazmeen made new friends and looked forward to going and socialising as well as participating in the support group. Nazmeen reported that the support given by the Health Action Local Engagement (HALE) Community Connector was excellent and Nazmeen really appreciated the help to move forwards.<br><br>Long-term impact: Eight months later Nazmeen was attending the group on a weekly basis and expressed an interest in volunteering somewhere where she could support people experiencing similar problems to hers. Nazmeen is now supporting a new fibromyalgia group set up by HALE and Champions Show the Way as a volunteer. Nazmeen feels like she is giving back to the community after all the support she received and says that volunteering has improved her self-confidence, self-esteem and life satisfaction.
National Association of Primary Care (2017)[7]	Not reported	"I am thrilled we tried it. Reading Well Books on Prescription scheme is currently used by the two pain nurses and our database shows that over six months we have

		issued more than 40 prescriptions. Whether a patient gets a prescription is reliant on us determining that this is an appropriate treatment option for the person in front of us and them agreeing to it. I have found that patients have been surprised and intrigued by being offered a book ‘prescription’; it feels reassuringly ‘medical’. Even as a pilot scheme, Reading Well Books on Prescription has made a difference in South Devon and I am thrilled we tried it. One patient who accessed Overcoming Chronic Pain said she found it helpful and commented that she is now aware that her pain ‘may well get worse but won’t kill’ her. She was very interested in the chapter on exercise and was delighted to find that she can still touch her toes, which she demonstrated to me in the clinic. She understands that exercise is safe and requested details about a ‘sensible exercise’ programme. Before being prescribed the book, this lady had a very medical focus; she wanted surgery. She now believes that she can develop self-management skills without having to rely on the NHS.” Dr Dee Burrows, Clinical Nurse Specialist in Chronic Pain
Sheffield Clinical Commissioning Group (2019) [8]	Not reported	“I have a bit of depression so I was quiet at first but I talk now because it’s so relaxed and calm”<br>[Participant from the Chronic Pain Group, Southey and Owlerton Area Regeneration (SOAR) community regeneration charity]<br><br>“My Sciatica has been eradicated; I couldn’t stand for even 5 minutes but now can walk for 1.5 hours a week” Health Walk, Darnall Wellbeing

1. Wright E, Zarnegar R, Hermansen I, et al. A clinical evaluation of a community-based rehabilitation and social intervention programme for patients with chronic pain with associated multi-morbidity. *Journal of Pain Management* 2017; 10: 149-159. Article.

2. Haake S, Bullas A and Quirk H. parkrun and the promotion of physical activity: insights for primary care clinicians from an online survey. *British Journal of General Practice* 2022; 72: E634-E640. Article. DOI: 10.3399/BJGP.2022.0001.

3. Corline A, Cole F, Trewern L, et al. 'Power to the People, to the people': Training for social prescribers improves support of persistent pain. *Br J Pain* 2023; 17: 281-292. DOI: 10.1177/20494637231152979.

4. Dayson C and Bashir N. *The social and economic impact of the Rotherham Social Prescribing Pilot: Main Evaluation Report*. 2014. Centre for Regional Economic and Social Research (CRESR).

5. Dayson C. Evaluation of HALE community connectors social prescribing, https://www.shu.ac.uk/-/media/home/research/cresr/reports/e/eval-hale-community-connectors-social-prescribing.pdf (2018).
6. Dayson C and Leather D. *Evaluation of HALE Community Connectors Social Prescribing Service 2018-19*. 2020. Centre for Regional Economic and Social Research (CRESR).
7. National Association of Primary Care (NAPC). *Books on Prescription How bibliotherapy can help your patients and save your practice time and money*. 2017. National Association of Primary Care (NAPC).
8. Sheffield Clinical Commissioning Group. People Keeping Well in their Community. 2019.
